# Restoration of p16^INK4A^ protein induces myogenic differentiation in RD rhabdomyosarcoma cells

**DOI:** 10.1038/sj.bjc.6690165

**Published:** 1999-03

**Authors:** M Urashima, G Teoh, M Akiyama, Y Yuza, K C Anderson, K Maekawa

**Affiliations:** Department of Pediatrics, Jikei University School of Medicine, Tokyo, Japan; Centre for Hematologic Oncology, Dana-Farber Cancer Institute and Department of Medicine, Boston, MA, USA

**Keywords:** p16, rhabdomyosarcoma differentiation, cell cycle

## Abstract

p16^INK4A^ (p16) tumour suppressor induces growth arrest by inhibiting function of cyclin-dependent kinase (CDK)4 and CDK6. Homozygous *p16* gene deletion is frequent in primary rhabdomyosarcoma (RMS) cells as well as derived cell lines. To confirm the significance of *p16* gene deletion in tumour biology of RMS, a temperature-sensitive *p16* mutant (E119G) gene was retrovirally transfected into the human RMS cell line RD, which has homozygous gene deletion of *p16* gene. Decrease from 40°C (restrictive) to 34°C (permissive) culture temperature reduced CDK6-associated kinase activity and induced G1 growth arrest. Moreover, RD-p16 cells cultured under permissive condition demonstrated differentiated morphology coupled with expressions of myogenin and myosin light chain. These suggest that deletion of *p16* gene may not only facilitate growth but also inhibit the myogenic differentiation of RD RMS cells. © 1999 Cancer Research Campaign


*British Journal of Cancer* (1999) **79**(7/8), 1032–1036
© 1999 Cancer Research Campaign
Article no. bjoc.1998.0165

### Restoration of p16$^{INK4A}$ protein induces myogenic differentiation in RD rhabdomyosarcoma cells

**M Urashima[1], G Teoh[2], M Akiyama[1], Y Yuza[1], KC Anderson[2] and K Maekawa[1]**

[1]Department of Pediatrics, Jikei University School of Medicine, 3-25-8, Nishi-shinbashi, Minato-ku Tokyo, 105-8461, Japan; and [2]Centre for Hematologic Oncology, Dana-Farber Cancer Institute and Department of Medicine, Harvard Medical School, Boston, MA, USA

**Summary** p16$^{INK4A}$ (p16) tumour suppressor induces growth arrest by inhibiting function of cyclin-dependent kinase (CDK)4 and CDK6. Homozygous *p16* gene deletion is frequent in primary rhabdomyosarcoma (RMS) cells as well as derived cell lines. To confirm the significance of *p16* gene deletion in tumour biology of RMS, a temperature-sensitive *p16* mutant (E119G) gene was retrovirally transfected into the human RMS cell line RD, which has homozygous gene deletion of *p16* gene. Decrease from 40°C (restrictive) to 34°C (permissive) culture temperature reduced CDK6-associated kinase activity and induced G1 growth arrest. Moreover, RD-p16 cells cultured under permissive condition demonstrated differentiated morphology coupled with expressions of myogenin and myosin light chain. These suggest that deletion of *p16* gene may not only facilitate growth but also inhibit the myogenic differentiation of RD RMS cells.

**Keywords:** p16; rhabdomyosarcoma differentiation; cell cycle

Rhabdomyosarcoma (RMS) is a childhood malignant tumour originating from immature mesenchymal cells that rarely demonstrate myogenic differentiation (Carli et al, 1992). Alveolar RMS has been associated with a characteristic translocation, t(2;13)(q35;q14) (Turc-Carel et al, 1986), which juxtaposes the *PAX3* gene known to regulate transcription during early neuromuscular development to the *FKHR* gene, a member of the forkhead family of transcription factors (Shapiro et al, 1993). Embryonal RMS is associated with loss of heterozygosity (LOH) at the 11p15 locus (Scrable et al, 1987), which affects the expression of insulin-like growth factor (IGF), a growth factor of RMS (El-Badry et al, 1990). Although much has been learned in the past decade regarding molecular genetic alterations associated with the development of RMS, exact mechanisms for aberrant growth without muscle differentiation are still obscure.

*p16$^{INK4A}$* (*p16*) gene induces dephosphorylation of pRB by inhibiting binding of cyclin-dependent kinase (CDK)4 and CDK6 to cyclin D, resulting in G1 growth arrest (Serrano et al, 1993). The discovery that *p16* gene is mutated or deleted in a striking proportion of human tumours raised the possiblity that abnormalities in *p16* might predispose to cancer development (Hirama et al, 1995). Of interest, homozygous deletion of *p16* gene was observed only in primary tumours of lymphoid malignancies (Ogawa et al, 1994; Hatta et al, 1995), although point mutations in *p16* gene were noted in many other kinds of tumours (Hussussian et al, 1994; Ranade et al, 1995). Moreover, *p16* gene deletion is frequently detected in primary tumours, whereas *p16* point mutations are associated with progressive cancer and established cell lines rather than primary tumours (Hirama et al, 1995). Therefore, *p16* gene deletion might be related with tumorigenesis; on the other hand, *p16* gene point mutations may contribute to disease progression. Frequent *p16* gene deletion has been reported in RMS cells, including 100% of cell lines and 25% of primary tumours (Iolascon et al, 1996), as in lymphoid malignancies, suggesting its potential importance in tumorigenesis of RMS.

To confirm the significance of *p16* gene deletion in tumour biology of RMS, *p16* gene was retrovirally transfected into the human RMS cell line RD, which has homozygous gene deletion of *p16* gene. Since ectopic expression of *p16* suppresses cell growth, a temperature-sensitive *p16* mutant (E119G) was used in this experiment. Restoration of functional p16 protein induced not only G1 growth arrest but also myogenic differentiation evidenced by morphological changes and expressions of myogenin and myosin light chain in RD–RMS cells. Our data, therefore, suggest that deletion of the *p16* gene not only facilitates growth but also inhibits myogenic differentiation of RD–RMS cells.

*Received 20 March 1998*
*Revised 16 September 1998*
*Accepted 22 September 1998*

*Correspondence to:* M Urashima

### MATERIALS AND METHODS

### Cell line and culture

RD, a cell line established from a patient with embryonal RMS (DeGiovanni et al, 1989), was purchased from American Type Culture Collection (Rockville, MD, USA) and maintained in Dulbecco's modified Eagle medium (DMEM; Gibco, Grand Island, NY, USA) supplemented with 10% fetal bovine serum (FBS; Gibco), 100 U ml$^{-1}$ penicillin (Gibco), 20 mM L-glutamine, and 100 μg ml$^{-1}$ streptomycin (Gibco) in humidified air with 5% dioxygen and 5% carbon dioxide at 37°C. For differentiation, RD cells were cultured in 2% horse serum plus DMEM.

### Polymerase chain reaction

DNA was isolated from RD cells as previously described (Urashima et al, 1996*a*). Polymerase chain reaction (PCR) was performed on an OmniGene Thermocycler (Marsh Biomedical, Rochester, NY, USA) with 100 ng of genomic DNA, 40 pM of sense and antisense primers, 200 mM each of dNTP, 1 × amplification buffer, 1.5 mM magnesium chloride 0.5 ml (2.5 units) *Taq* polymerase and 10% dimethyl sulfoxide in a reaction volume of 25 μl.

Amplification consisted of 94°C for 2 min, followed by 30 cycles of denaturation at 94°C for 30 s, annealing at 55°C for 1 min, and extension at 72°C for 1 min. Primers for amplifying p16 exon 2 were 5′-GCT TCC **TTT CCG** TCA TGCCG-3′ and 5′-GGA CTG ATG ATC ATG GCT CCA CCT GCCTT-3′. As a control, β-globin sequences were amplified using the following oligonucleotides: sense primer 5′-AAC AGA CAC CAT GGT GCA CC-3′, and antisense primer 5′-CTA AGG TGA AGG CTC ATG GC-3′. The resulting PCR products were electrophoresed on an ethidium bromide-stained 3.0% agarose gel. The size of p16 and β-globin products are 393 base pairs (bp) and 362 bp, respectively.

### Mutagenesis

The construction of *p16* mutants was carried out with BioRad Muta-gene Phagemid in vitro Mutagenesis System using full-length p16 cDNA including exons 1α, 2 and 3 [provided by Dr Geoffrey I. Shapiro, Dana-Farber Cancer Institute (DFCI), Boston, MA, USA] Complementary DNA strand for mutated *p16* gene (E119G) was made using a synthetic oligonucleotide (5′GATG-GCCCAGCTCGCCGGCCAGGTCCACGG3′) as primer, followed by cloning into EcoRI/SalI site of pBabe-puro retroviral vector (provided by Dr Mark Ewen, DFCI). Of the mutations attempted in previous study, a mutation at position 119 (E119G) was found to be restrictive at higher temperature and permissive at lower temperature for binding to CDK4 and CDK6, inhibiting CDK4 and CDK6 complex kinase activities, decreasing phosphorylation of pRB, and inhibiting growth (Urashima et al, 1997*c*).

### Production of retrovirus and transfection pBabe-puro (control) and pBabe-p16 mutated type (E119G) vector were introduced into Bing packaging cells, obtained from Dr Shapiro (DFCI), using standard calcium phosphate transfection technique (Morgenstern and Land, 1990). Bing cells were cultured for 1-day post-transfection in DMEM with 10% FBS, and supernatant was exchanged with fresh media for an additional 2 days. Retroviral supernatants were then harvested post-transfection, and filtered through a 0.45 μm filter to remove living cells. RD–RMS cells were cultured on a 100 mm tissue culture plate for 18 h prior to infection. Supernatant was exchanged with 3 ml infection cocktail consisting of fresh retroviral-containing supernatant and polybrene at a final concentration of 4.0 μg $ml^{-1}$ (Sigma, St Louis, MO, USA) for 3 h, followed by addition of fresh media (7 ml). Selection for mutated *p16* gene transdused RD (RD-p16) cells and control vector transfectant (RD-control) was performed by culture with puromycin (2.0 μg $ml^{-1}$)(Sigma) (Urashima et al, 1997*a*). A colony expressing the highest level of p16 protein was selected at 2 weeks and amplified for a further 2 weeks at 40°C.

### Immunoprecipitation and Western blotting

Immunoprecipitation (IP) and Western blotting (WB) were performed as previously described (Urashima et al, 1996*b*). For IP, cells (2 × $10^6$ cells/sample) were washed thrice with phosphate-buffered saline (PBS) and lysed for 30 min at 4°C in buffer: 1 mM Tris-HCl (pH 7.6), 150 mM sodium chloride, 0.5% Nonidet p-40, 5 mM EDTA, 1 mM phenylmethylsulphonyl fluoride, sodium phosphate (v), aprotinin, and 1 mM NaF. Anti-p16 monoclonal antibody (Ab) (Pharmigen, San Diego, CA, USA); anti-CDK6 polyclonal Ab (Santa Cruz Biotechnology, Santa Cruz, CA, USA) were added for 16 h at 4°C to immunoprecipitate protein complexes. Proteins were collected using protein G sepharose (PGS). Aliquots of each lysate were analysed by sodium dodecyl sulfate-polyacrylamide gel electrophoresis (SDS–PAGE). Proteins were transferred onto PVDF membrane (NEN Dupont, Boston, MA, USA), and non-specific binding was blocked by incubation with 5% skim milk. The membrane was probed with Ab followed by anti-mouse or anti-rabbit Ig Abs conjugated with horseradish peroxidase (HRP; Amersham, Arlington Heights, IL, USA). Complexes were detected using the enhanced chemiluminescence system (Amersham). To characterize differential stage of RD cells, IP and WB were performed using anti-p18, anti-p21, anti-myogenin polyclonal Abs (Santa Cruz Biotechnology); anti-Myo D monoclonal Ab (Santa Cruz Biotechnology); anti-myosin light chain monoclonal Ab (Sigma).

### Immune complex kinase assays

Immune complex kinase assays were performed as previously described with modification (Urashima et al, 1997*b*). Briefly, cells (1 × $10^7$ $ml^{-1}$) were suspended in lysis buffer, centrifuged, and supernatants precipitated for 16 h at 4°C with PGS plus rabbit anti-CDK6 polyclonal Ab. Immunoprecipitated proteins on PGS were washed thrice with 1 ml of lysis buffer and twice with 50 mmol $l^{-1}$ HEPES (pH 7.5) containing 1 mmol l dithiothreitol and suspended in 30 μl of kinase buffer (50 mmol $l^{-1}$ HEPES, 10 mmol $l^{-1}$ $MgCl_2$, $1^{-1}$ mmol l dithiothreitol) containing substrate and 2.5 mmol $l^{-1}$ EGTA, 10 mmol $l^{-1}$ β-glycerophosphate 0.1 mmol $l^{-1}$ sodium orthovanadate, 1 mmol $l^{-1}$ NaF, 20 μmol $l^{-1}$ adenosine 5′-triphosphate (ATP), and 10 μCi of $\gamma$-$^{32}$P-ATP (NEN Dupont, Boston, MA, USA). For CDK6 kinase assays, 1 μg of soluble glutathione S-transferase (GST)–RB fusion protein (Santa Cruz Biotechnology) was used as the substrate. After incubation for 30 min at 30°C with occasional mixing, the samples were boiled in polyacrylamide gel sample buffer and separated by SDS electrophoresis. Phosphorylated proteins were visualized by autoradiography of the dried slab gels.

### Cell cycle analysis

Cell cycle distribution of RD cells was examined using propidium iodide (PI; Sigma) staining and FACS analysis, as in a previous report (Urashima et al, 1997*c*). Briefly, cells were collected and suspended in 0.5 ml of 3.4 mM sodium citrate, 10 mM NaCl, 0.1% NP-40 and 50 ng $ml^{-1}$ PI to stain nuclear DNA. Cell cycle distribution for each sample (> 10 000 cells) was determined using the flow cytometer (Ortho-Clinical Diagnostics KK, Koto-ku, Tokyo, Japan).

### RESULTS

### Homozygous deletion of *p16* gene and ectopic expression of p16 protein in RD RMS cells

We first confirmed homozygous deletion of the *p16* gene (exon 2) in RD–RMS cells using mixed primers for exon 2 of the *p16* gene and β-globin gene in a PCR assay. As can be seen in Figure 1, p16 exon 2 was not detectable in genomic DNA of RD–RMS cells by

© Cancer Research Campaign 1999

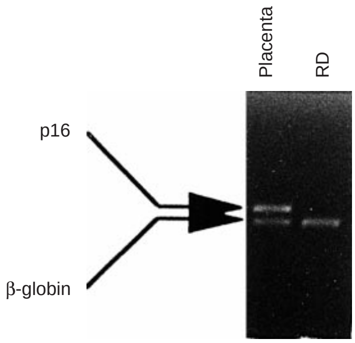

**Figure 1** Homozygous deletion of *p16* gene in RMS cells. PCR was performed using extracted DNA from control placenta and RD cells with amplification using primers for p16 exon 2 and β-globin genes. The resulting products were electrophoresed on an ethidium bromide-stained 3.0% agarose gel. The sizes of p16 and β-globin PCR products were 393 bp and 362 bp, respectively

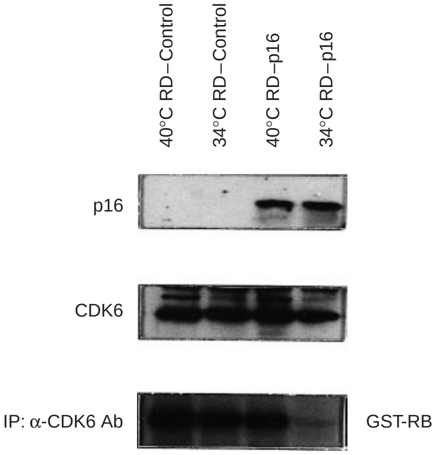

**Figure 2** Ectopic expression of p16 protein in RMS cells and effects of culture temperature on CDK6. RD cells were transfected with control vector or mutated *p16* gene, and transfectants were selected by culture with puromycin for 4 weeks. Expressions of p16 protein and CDK6 in RD–control (cont.) cells and RD–p16 cells cultured at 40°C or 34°C for 24 h, were evaluated by IP and WB. Cell lysates were precipitated for 16 h with PGS plus anti-CDK6 Ab. Immunoprecipitated proteins on PGS were resuspended in kinase buffer after washing. GST–RB fusion protein was used as a substrate for assay of CDK6-associated kinase activity

**A**

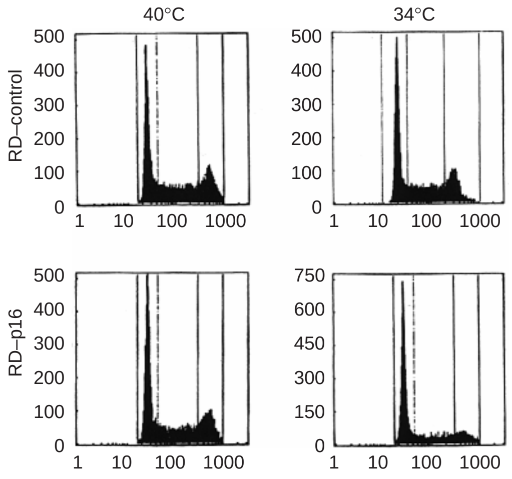

| | RD–Control | | RD–p16 | |
|---|---|---|---|---|
| | 40°C | 34°C | 40°C | 34°C |
| $G_0/G_1$ | 47 | 49 | 51 | 73 |
| S | 35 | 34 | 32 | 19 |
| $G_2M$ | 18 | 17 | 17 | 8 |

Unit=%

**B**

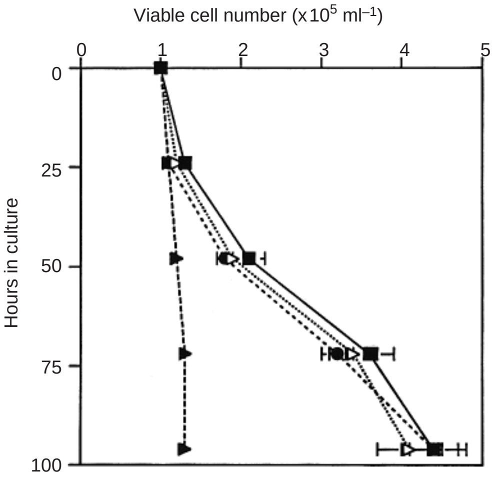

**Figure 3** Effect of ectopic p16 expression on proliferation of RD cells. (**A**) RD–control (cont.) cells (1 × $10^5$ cells/ml) and RD–p16 cells (1 × $10^5$ cells/ml) incubated with puromycin (2 μg ml$^{-1}$) for 4 weeks were cultured in fresh media including puromycin (2 μg ml$^{-1}$) either at 40°C or 34°C for 72 h. Cell cycle distribution was determined using PI staining followed by flow cytometric analysis. (**B**) RD-control cells were cultured at either 40°C (■) or 34°C (△) and RD–p16 cells were cultured at either 40°C (●) or 34°C (▲) for 96 h. Viable cell number was assessed by trypan blue staining

PCR, whereas it was present in control cells from placenta. The β-globin gene served as control.

Once we confirmed homozygous deletion of *p16* gene in RD cells, a temperature-sensitive mutated *p16* gene (E119G) was ectopically expressed in RD cells by retroviral transfection. p16 protein was expressed in the *p16* gene transfected RD (RD–p16) cells at either 40°C or 34°C, but it was not detectable within control vector transfected RD (RD–control) cells at either 40°C or 34°C (Figure 2). Expression of CDK6 protein was equivalent in these cells. However, activity of CDK6 was inhibited in cell lysates of RD-p16 cells cultured at 34°C compared with RD-control cells and RD–p16 cells cultured at 40°C.

### Effect of functional p16 protein on proliferation of RD cells

RD-control and RD-p16 cells were cultured for 72 h at either 40°C or 34°C, and cell cycle distribution was determined by PI staining (Figure 3A). At 40°C, the percentage of cells in G1 phase was similar in RD–control cells (47%) and RD–p16 cells (51%). In contrast, the percentage of RD-p16 cells in G1 phase at 34°C increased to 73%, although cell cycle distribution of RD–control cells did not significantly vary with culture temperature. Moreover, sub G0 population including apoptotic and dead cells was not observed in all conditions. Viable cell number did not

 *© Cancer Research Campaign 1999*

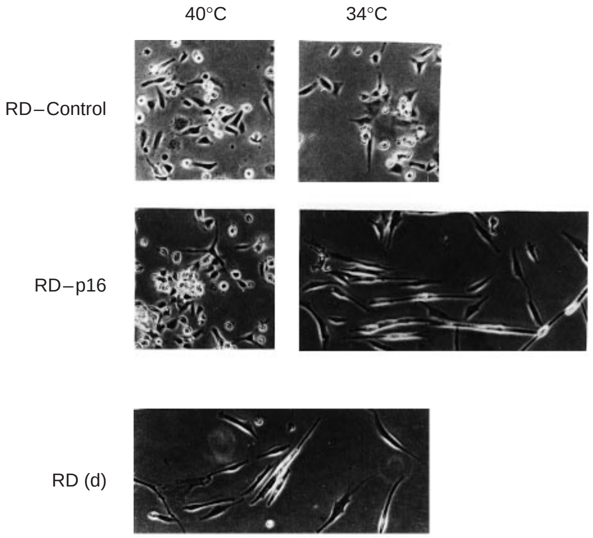

**Figure 4** Effect of functional p16 expression on morphology of RD cells. RD–control (cont.) and RD–p16 cells were cultured at either 40°C or 34°C for 7 days. As a control, RD cells cultured in differentiation medium (2% horse serum) for 7 d (RD (d)) at 37°C. Morphological changes were observed under phase contrast microscopy. Original magnification was × 100

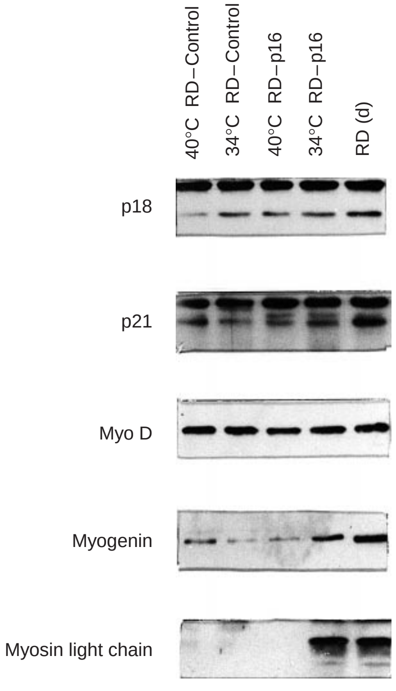

**Figure 5** Effect of ectopic p16 expression on differentiation of RD cells. Expressions of p18, p21, Myo D, myogenin, and myosin light chain protein in RD–control (cont.) and RD–p16 cells cultured at either 40°C or 34°C for 7 days as well as RD cells cultured in differentiation medium (2% horse serum) for 7 days (RD (d)) were determined by IP and WB

increase in RD–p16 cells cultured at 34°C, but increased in RD–control cells cultured at 40°C and 34°C and RD–p16 cells cultured at 40°C (Figure 3B). Percentages of dead cells counted with trypan blue were less than 10% in all conditions.

### Effect of functional p16 protein on morphology and differentiation of RD cells

Under phase contrast microscopy, RD–p16 cells cultured at 34°C demonstrated a differentiated morphology, with elongated myotube-formation and multinuclei, whereas RD–p16 cells cultured at 40°C, as well as RD–control cells cultured at 40°C and 34°C, demonstrated round form or fibroblastic morphology (Figure 4). Although RD cells cultured in differentiation medium (2% horse serum) at 37°C showed differentiation tendency (Figure 4), effect of active p16 expression at 34°C did not enhance the differentiation phenotype significantly (data not shown).

RD–p16 cells were cultured under either restrictive (40°C) or permissive (34°C) condition for 7 d and RD cells were cultured in differentiation media for 7 d to examine for expressions of p18, p21, Myo D, myogenin, and myosin light chain proteins (Figure 5). Expressions of p18, p21, and Myo D proteins were unchanged in RD–control cells and RD–p16 cells cultured at either 40°C or 34°C, and were lower than in RD cells cultured in differentiation medium. On the other hand, expression level of myogenin protein was low in RD–control cells cultured at either 40°C or 34°C and RD–p16 cells cultured at 40°C, whereas it was significantly higher in RD–p16 cells cultured at 34°C and in RD cells cultured in differentiation medium. Myosin light chain protein was also strongly expressed in RD–p16 cells cultured at 34°C and RD cells cultured in differentiation medium, whereas it was not detectable in RD–control cells cultured at either 40°C or 34°C and RD–p16 cells cultured at 40°C.

### DISCUSSION

In the present report, we demonstrated that lack of *p16* gene may both facilitate cell proliferation and inhibit myogenic differentiation using RD–RMS cells. We first confirmed homozygous deletion of p16 gene exon 2 in RD cells by PCR, and lack of p16 protein by IP and WB. An alternative RNA transcript for p16 (p16 β) has been identified (Mao et al, 1995; Stone et al, 1995), and is composed of exon 1β, upstream from exon 1α of p16, spliced onto the remaining exons 2 and 3 of p16. Although p16β transcript was not studied in this experiment, homozygous deletion of p16 exon 2 may suggest lack of the p16β transcript. Next p16 protein was expressed ectopically in RD–RMS cells using retroviral transfection in order to investigate the role of homozygous deletion of *p16* gene in growth and differentiation of RMS cells. However, growth inhibition in RD cells ectopically expressing p16, coupled with the outgrowth of clones that express low levels of this protein, complicate this approach. To overcome this limitation, we attempted to create p16 temperature-sensitive mutants (Urashima et al, 1997*c*).

In previous reports, function of p16 is believed to associate with growth inhibition, cell mortality and senescence (Serrano et al, 1996, 1997). However, in this study, restoration of functional p16 protein in RD cells also induced myogenic differentiation, associated with morphological changes and with expression of myosin light chain protein, which is specifically expressed in differentiated myocytes and syncytial myotubules but not in proliferating myoblasts (Andres and Walsh, 1996). Moreover, p21 is expressed low in proliferating myoblasts but high in differentiated myocytes (Havery et al, 1995; Parker et al, 1995). In this study, p21 and p18 expression increased slightly in RD cells cultured in differentiation medium, whereas they were not altered in RD cells with

© Cancer Research Campaign 1999

activated p16. In a normal murine myoblast model, myogenic differentiation was inhibited by forced expression of cyclin D1; in contrast, myoblast differentiation was enhanced by transfection with p16 (Skapek et al, 1995). p16, therefore, may be associated with normal myogenesis and lack of *p16* gene may contribute to development of RMS cells. Retinoblastoma protein, which is a specific target of p16, interacts with Myo D family to induce myogenic differentiation (Gu, 1993). In addition, myotubes from retinoblastoma gene (–/–) cells cannot withdraw from cell cycle (Schneider et al, 1994), suggesting retinoblastoma gene product is required for permanent withdrawal from cell cycle and late stage of muscle differentiation (Novitch et al, 1996). Lack of p16 in RMS cells may lead to inactivation of retinoblastoma protein, resulting in aberrant growth and defect of terminal differentiation.

### ACKNOWLEDGEMENT

This study was funded by Osaka Gan Kenkyu.

### REFERENCES

Andres V and Walsh K (1996) Myogen expression, cell cycle withdrawal, and phenotypic differentiation are temporally separable events that precede cell fusion upon myogenesis. *J Cell Biol* **132**: 657–666

Carli M, Guglielmi M, Sotti G, Cecchetto G and Ninfo V (1992) Rhabdomyosarcoma. In *Paediatric Oncology*; Plowman PN and Pinkerton CR (eds), pp. 291–324. Chapman & Hall Medical: New York

DeGiovanni C, Nanni P, Nicoletti G, Ceccarelli C, Scotland K, Landuzzi L and Lollini PL (1989) Metastatic ability and differentiative properties of a new cell line of human embryonal rhabdomyosarcoma (CCA). *Anticancer Res* **9**: 1943–1950

El-Badry OM, Minniti C, Kohn EC, Houghton PJ, Daughaday WH and Helman LJ (1990) Insulin-like growth factor II acts as an autocrine growth and motility factor in human rhabdomyosarcoma tumors. *Cell Growth Differ* **1**: 325–331

Gu W (1993) Interaction of myogenic factors and the retinoblastoma protein mediates muscle cell commitment and differentiation. *Cell* **72**: 309–324

Hatta Y, Hirama T, Miller CW, Yamada Y, Tomonaga M and Loeffler HP (1995) Homozygous deletion of the p15(MTS2) and p16(CDKN2/MTS1) genes in adult T-cell leukemia. *Blood* **85**: 2699–2740

Havery O, Novitch BG, Spicer DB, Skapek SX, Rhee J, Hannon GJ, Beach D, Lassar AB (1995) Correlation of terminal cell cycle arrest of skeletal muscle with induction of p21 by Myo D. *Science* **267**: 1018–1021

Hirama T and Koeffler HP (1995) Role of cyclin-dependent kinase inhibitors in the development of cancer. *Blood* **86**: 841–854

Hussussian CJ, Struewing JP, Goldstein AM, Higgins PAT, Ally DS, Sheathan MD, Clark Jr WH, Tucker MA and Dracopoli NC (1994) Germline p16 mutations in familial melanoma. *Nature Genet* **8**: 15–21

Iolascon A, Faienza MF, Coppola B, Rosolen A, Basso G, Della Ragione F and Schettini F (1996) Analysis of cyclin-dependent kinase inhibitor genes (CDKN2A, CDKN2B, and CDKN2C) in childhood rhabdomyosarcoma. *Genes Chrom Cancer* **15**: 217–222

Mao L, Merlo A, Bedi G, Shapiro GI, Edwards CD, Rollins BJ and Sidransky D (1995) A novel $p16^{INK4A}$ transcript. *Cancer Res* **55**: 2995–2997

Morgenstern JP and Land H (1990) Advanced mammalian gene transfer: high titre retroviral vectors with multiple drug selection markers and a complementary helper-free packaging cell line. *Nucleic Acids Res* **18**: 3587–3596

Novitch BG, Mulligan GJ, Jacks T and Lassar AB (1996) Skeletal muscle cells lacking the retinoblastoma protein display defects in muscle gene expression and accumulation in S and G2 phase of the cell cycle. *J Cell Biol* **135**: 491–456

Ogawa S, Hirano N, Sato N, Takahashi T, Hangaishi A, Tanaka K, Kurokawa M, Tanaka T, Mitani K, Yazaki Y and Hirai H (1994) Homozygous loss of the cyclin-dependent kinase 4-inhibitor (p16) gene in human leukemias. *Blood* **84**: 2431–2435

Parker SB, Eichele G, Zhang P, Rawls S, Sands AT, Bradley A, Olson EN, Harper JW and Elledge SJ (1995) p53-independent expression of p21CIO1 in muscle and other terminally differentiating cells. *Science* **267**: 1024–1027

Ranade K, Hussussian CJ, Sikorski RS, Varmus HE, Goldstein AM, Tucker MA, Serrano M, Hannon GJ, Beach D and Dracopoli NC (1995) Mutation associated with familial melanoma impairs $p16^{INK4}$ function. *Nature Genet* **10**: 114–116

Schneider JW, Gu W, Zhu L, Mahdavi V and Nadal-Ginard B (1994) Reversal of terminal differentiation mediated by p107 in Rb –/– muscle cells. *Science* **264**: 1467–1471

Scrable HJ, Witte DP, Lampkin BC and Cavenee WK (1987) Chromosomal localization of the human rhabdomyosarcoma locus by mitotic recombination mapping. *Nature* **329**: 645–647

Serrano M, Hannon GJ and Beach D (1993) A new regulatory motif in cell cycle control causing specific inhibition of cyclin D/CDK4. *Nature* **366**: 704–707

Serrano M, Lee H-W, Chin L, Cordon-Cardo C, Beach D and DePinho RA (1996) Role of the INK4a locus in tumor suppression and cell mortality. *Cell* **85**: 27–37

Serrano M, Liu AM, McCurrach ME, Beach D and Lowe SW (1997) Oncopenic ras provokes premature all senescence associated with accumulation of p53 and $p16^{INK4a}$. *Cell* **88**: 593–602

Skapek SX, Rhee J, Spicer DB and Lassar AB (1995) Inhibition of myogenic differentiation in proliferating myoblasts by cyclin D1-dependent kinase. *Science* **267**: 1022–1024

Shapiro DN, Sublett JE, Li B, Downing JR and Naeve CW (1993) Fusion of Pax3 to a member of the fork-head family of transcription factors in human alveolar rhabdomyosarcoma. *Cancer Res* **53**: 5108–5112

Stone S, Jiang P, Dayananth P, Tavtigian SV, Katcher H, Parry D, Peters G and Kamb A (1995) Complex structure and regulation of the p16 (MTSl) locus. *Cancer Res* **55**: 2988–2994

Turc-Carel C, Lizard-Nacol S and Justrabo E (1986) Consistent chromosomal translocation in alveolar rhabdomyosarcoma. *Cancer Genet Cytogenet* **19**: 361–362

Urashima M, Hoshi Y, Sugimoto Y, Kaihara C, Matsuzaki M, Chauhan D, Ogata A, Teoh G, DeCaprio JA and Anderson KC (1996*a*) A novel pre-B acute lymphoblastic leukemia cell line with chromosomal translocation between $p16^{INK4A}/p15^{INK4B}$ tumor suppressor and immunoglobulin heavy chain genes. *Leukemia* **10**: 1576–1583

Urashima M, Ogata A, Chauhan D, Vidriales MB, Teoh G, Hoshi Y, Schlossman RL, DeCaprio JA and Anderson KC (1996*b*) Interleukin-6 promotes multiple myeloma cell growth via phosphorylation of retinoblastoma protein. *Blood* **88**: 2219–2227

Urashima M, Teoh G, Ogata A, Chauhan D, Treon SP, Sugimoto Y, Kaihara C, Matsuzaki M, Hoshi Y, DeCaprio JA and Anderson KC (1997*a*) Characterization of $p16^{INK4A}$ expression in multiple myeloma and plasma cell leukemia. *Clin Cancer Res* **3**: 2173–2179

Urashima M, Teoh G, Chauhan D, Hoshi Y, Ogata A, Treon SP, Schlossman RL and Anderson KC (1997*b*) Interleukin-6 overcomes $p21^{WAF1}$ upregulation and G1 growth arrest induced by dexamethasone and interferon $\gamma$ in multiple myeloma cells. *Blood* **90**: 279–289

Urashima M, DeCaprio JA, Teoh G, Ogata A, Chauhan D, Treon SP, Hoshi Y and Anderson KC (1997*c*) $p16^{INK4A}$ promotes differentiation and inhibits apoptosis of JKB acute lymphoblastic leukemia cells. *Blood* **90**: 4106–4115

 *© Cancer Research Campaign 1999*
